# Neurotransmitter Assay for In Vivo Nerve Signal Detection Using Bismuth Immobilized on a Carbon Nanotube Paste Electrode

**DOI:** 10.3390/mi14101899

**Published:** 2023-10-02

**Authors:** Jongwan Choi, Jiwon Min, Jason Sahngwook Kim, Jung Hyun Park, SuwYoung Ly

**Affiliations:** 1 Department of Chemistry and Life Science, Sahmyook University, Seoul 01795, Republic of Korea; jchoi@syu.ac.kr; 2 College of Pharmacy, University of Rhode Island, Kingston, RI 02881, USA; 3 Biosensor Research Institute, Seoul National University of Science &Technology, Seoul 01811, Republic of Korea

**Keywords:** neurotransmitter, bismuth, carbon nanotube, voltammetry, biomedical engineering

## Abstract

Background: Voltammetric analysis of the neurotransmitter epinephrine (EP) was performed using bismuth immobilized on a carbon nanotube paste electrode (BCE), whose properties were compared with those of a carbon nanotube paste electrode (CE). BCE was found to be more efficient in detecting EP. Methods: The analytical parameters used were 0.3 V square-wave (SW) stripping voltammetric amplitude, 400 Hz frequency, −0.8 V initial potential, and 0.015 V increment potential. The optimized conditions were applied to an assay of a carp’s front fin. Results: A BCE was inserted into a carp’s front fin muscle, and a stimulus was given every 50 s. This circuit is easy to use and does not require much analytical preparation time. Conclusions: The working electrode is miniscule, and its detection limit is very low. The in vivo muscle’s chronoamperometric nerve currents were analyzed. These results have potential for applications in medical diagnostics, pharmaceuticals, interface controllers, and other fields.

## 1. Introduction

The neurotransmitter epinephrine, which is also a hormone, is secreted by the adrenal medulla. It serves as a chemical mediator for conveying nerve pulses to different organs [1]. EP also excites the sympathetic nerves and makes the heart beat fast. It also controls blood sugar and affects muscle control. 

The measurement of neurotransmitters is important in medicine, neuropsychiatric science, and other analytical fields. Thus, various methods for analyzing EP and its analogs have been developed. However, most of these methods were developed in laboratory conditions only. These methods employ high-performance liquid chromatography [2], capillary electrophoresis [3], reactors [4], and microelectrodes [5]. Moreover, these methods are more complicated than the electroanalytical method and are not suitable for in vivo applications or analytical trace detection ranges.

More recently, electroanalytical methods were used with some methods that exhibit low detection limits. Examples of these are carbon fiber ultra-micro electrodes (detection limit: 7.8 ng m/L) [6], modified glassy carbon electrodes (detection limit: 8 × 10^−9^ M) [7], and carbon fiber microelectrodes (detection limit: 0.009 mg/L) [8]. Although these electrodes have a low detection limit, they are more complicated to fabricate and are not suitable for use in direct in vivo assays. The proposed electrode, however, can be conveniently fabricated, although it is smaller than most electrodes. It is so tiny that it can be inserted into living body systems without destroying them.

Traditionally, mercury electrodes have been employed to achieve high reproducibility and sensitivity of the stripping technique, but because of the toxicity of mercury, new alternative electrode materials are much desired, particularly for on-site environmental monitoring of trace pollutants [9]. Bismuth, a semimetal with a rhombohedral structure, has been used for such purposes. Owing to its unique physical and chemical properties, it has been attracting much interest from physicists and chemists [10]. Bismuth is also nontoxic, unlike mercury, which is why it is being used instead of mercury. Some examples of uses of bismuth electrodes are bismuth film electrodes for adsorptive cathodic stripping analysis of trace nickel [11], deriving operational parameters for the advanced use of bismuth film electrodes [12], anodic stripping voltammetry with in situ bismuth-plated carbon and gold micro-disc electrodes [13], and bismuth film electrodes for anodic stripping voltammetric determination of tin and other metals [14,15]. These electrodes, however, are too big to be inserted into human or animal body systems. They are also likely to be destroyed upon insertion.

Moreover, a graphite carbon nanotube with a wide surface area was used because it could effectively serve as a catalyst [16,17]. It was used for immobilizing bismuth paste compositions. Here, the BCE is tiny and is macroscale. Objective-size three-cell systems could employ this electrode without destroying the organ tissue. This method can be applied in any field, including medical diagnostics, in vivo interfacing applications, and nerve current detection. 

Epinephrine causes the following redox body phenomena as a neurotransmitter: it is involved in muscle movement [18], and it generates electrical current signals [19] involved in processes such as metabolism, attention, concentration, fear, and excitement. It corresponds to sleep disorders, anxiety, high blood pressure, and decreased immunity. The main action of epinephrine is to act as a hormone. Therefore, in vitro amplification diagnosis of trace amounts of neurotransmitters becomes possible [20]. Here, it is secreted by the adrenal gland in response to stress. These reaction strengths can be diagnosed by measuring currents on the skin of the body muscle [21]. When the body is in danger, it generates a redox current of fight or flight. The fight-or-flight response generates a redox [22] muscle contraction current to block the danger. Also, when faced with fear, the human body generates tension currents [23], and when faced with a dangerous situation, the nerve current induces an escape signal to seek a safe place. The fight-or-flight response generates danger currents. These currents are diagnostic at the micro level [24]. 

When it detects a danger signal, the hypothalamic nerve sends an electrical current to the body below the spinal cord. Norepinephrine (noradrenaline) determines what action to undertake, and it sends a redox current message. The neurotransmitter noradrenaline sends electrical currents to muscles in organs and nervous tissue, and it causes a quick body reaction. The organs that can sense the currents and be measured are as follows [25]. Eyes: The human pupil dilates to let in more light so that we can see our surroundings better. Skin: Vascular muscles transport blood to areas that need more oxygen. Thus, when signaled to fight or flee, the skin contracts and turns pale. Heart: Muscle movement delivers more oxygenated blood to the areas that need it pumped quickly when needed. It also increases blood pressure. Muscles: Muscles receive more blood flow and oxygen and respond with greater force and speed. Liver: Glycogen stored in the liver is converted to glucose to provide more energy. Respiratory system: Breathing becomes deeper and faster, which increases the amount of oxygen. As the airways open, more oxygen passes into the blood and into the muscles [26].

Noradrenaline, a neuro transmitter, reaches the adrenal gland and produces adrenaline (epinephrine) and norepinephrine. This releases the hormone adrenaline (norepinephrine). These hormones travel to all parts of the body through the blood. These substances reach the eyes, heart, respiratory tract, skin blood vessels, and adrenal glands again. These organs and tissues continue to respond to the “message” until the danger is removed.

Next, nerves in an area of the brain called the hypothalamus send signals down the spinal cord, transmitting it to the body. Norepinephrine (noradrenaline) transmits the nervous system current to the brain. These redox nerve currents can be amplified and diagnosed [27,28,29,30].

## 2. Experimental Design

### 2.1. Preparation of the Electrode and In Vivo Implantation

Two types of BCE and CE [30] were used in this experiment. The first BCE working sensor was made with bismuth that was immobilized on a nanotube paste and mineral oil. The BCE was prepared by mixing 0.3 mL bismuth powder and 1 g carbon nanotube (the multiwalled carbon nanotube was obtained from Nanotech Co., Ltd., Choong Nam, Republic of Korea, Zip 330-816, by catalytic CVD; outside diameter: 15–40 nm; length: 30–50 μm. It was purified overnight prior to use by magnetic stirring in a 2 M nitric acid solution and washed using triple-distilled water) with mineral oil. The second CE was made by mixing nanotubes and mineral oil (50:50 w%) using the same method. The mixture was homogenized in a mortar for 30 min. The mixed paste was then inserted into a 2.0 mm × 5 cm long plastic needle-type capillary tube, and a 0.5 mm copper wire was connected to the electrochemical measurement system. An Ag/AgCl electrode and a 0.2 mm platinum wire electrode served as the reference and auxiliary electrode, respectively. A three-electrode cell was used to monitor the voltammetric signal. The in vivo implantation techniques were those used by previous studies [16,17].

### 2.2. Reagents

All experimental solutions were prepared from 18 M ohm cm^−1^ double-distilled water. The standard 1000 mg/L EP and other reagents were obtained at analytical grade (Aldrich, Saint Louis, MO, USA). All systems were carried out in dissolved oxygen, and the electrode reduction current was stable. Thus, cleaning time was not necessary for every measurement. The strength of the phosphate concentration effect was studied within a range of 0.01–0.5 M, and 0.1 M phosphate buffer was found to be the most suitable. Here, a 0.1 M NH_4_H_2_PO_4_ electrolyte solution with a pH level of 4.75 was found to be the most suitable medium (the pH 4.5 buffer solution was capable of buffering up to the pH range of 6.5~7.5 in the human or animal body). Experiments using animals or living tissues were approved by the Ministry of Korean Government Legislation Animal Ethics Code No. 7167, 2004, 2.9, following the Care and Use of Animals for Scientific Purposes guidelines.

### 2.3. Experimental Procedure

The common parameters for CV were a scan rate of 100 mVs^−1^, an initial potential of −2.0 V, and a switching potential of −2.0 V. The common parameters for SWSV were set at optimized conditions of 0.3 V SW amplitude, 400 Hz frequency, −0.8 V initial potential, 0.015 V increment potential, 1 V final potential, and 1 × 10^−4^ sensitivity. These were used at the 300 s accumulation time. Bismuth immobilization was performed through a cyclic scan with an initial potential of +1.6 V, a switching potential of −1.6 V, and a scan rate of 0.5 mVs^−1^, with a 10-cycle repetition to stabilize the electrode surface. Since the voltammetric response of EP depends on the electrolyte solutions and the hydrogen ionic strength, various types of electrolyte solutions were tested. The phosphate solution was found to have the best results.

## 3. Results and Discussion

### 3.1. Cyclic Voltammetry

**Figure 1:**
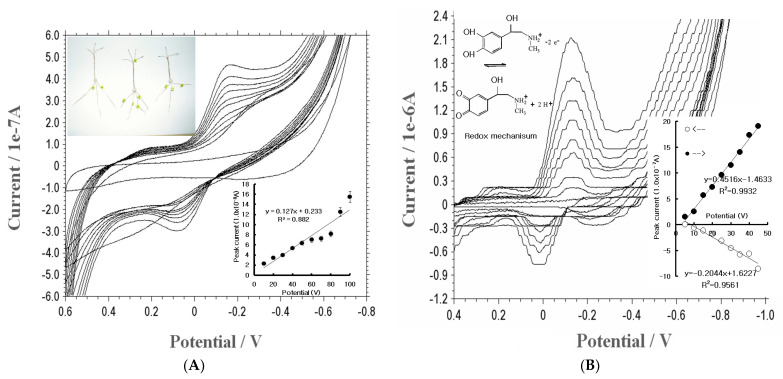
(**A**): Cyclic voltammetry for CE with −0.8 V initial potential, 0.6 V switching potential, 50 mV/s scan late, and concentrations of 10, 20, 30, 40, 50, 60, 70, 80, 90, and 100 mg/L EP variations. The inset curve is the oxidation peak current. (**B**) Cyclic voltammetry for BCE with EP concentrations of 5, 10, 15, 20, 25, 30, 35, 40, and 45 mg/L variations as in (**A**), using 0.1 M NH_4_H_2_PO_4_ electrolyte solution.

Using the same electrolyte solution, bismuth immobilization effects were compared on the BCE and CE working electrodes. The results showed that it was effective for EP detection. Figure 1A shows the CV using a CE electrode for various concentrations, from 10 to 100 mg/L spiked EP. Here, the oxidation and reduction peaks were obtained, and the inset curve is anodic for y = 0.127x + 0.233, and is thus not sensitive. Figure 1B, however, shows the electrical redox mechanism of CV using a BCE with a concentration from 5 to 45 mg/L. Under linear equations, the reduction peak was sensitive, and the oxidation peak current (19.8 × 10^−7^ A) increased twofold followed by anodic current (9.9 × 10^−7^ A). However, both curves are applicable in stripping voltammetry in EP analysis. The BCE reduction was also more sensitive than that of the CE, which is why a BCE was chosen as the working electrode [31]. In this solution, the more sensitive stripping voltammetric parameters were examined.

### 3.2. Optimization of the SW Parameters

**Figure 2:**
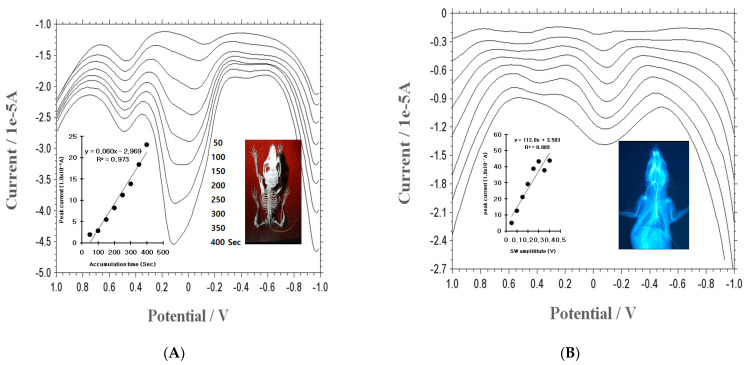
(**A**) Current responses in the SWSV accumulation time variations of 50, 100, 150, 200, 250, 300, 350, 400, 450, and 500 s. (**B**) Amplitudes of 0.05, 0.5, 0.15, 0.2, 0.25, 0.3, 0.35, and 0.4 V variations. Other conditions were used for the optimum conditions of 400 Hz frequency, −0.8 V accumulation forward potential, and 1.0 V backward potential, total 400 s accumulation time with the BCE.

The SWSV time variations were determined from 50 to 500 s, using a constant 30.0 mgL^−1^ EP concentration. As Figure 2A shows, the anodic current increased very quickly from 1.89 to 23.04 × 10^−6^ A. Here, the 400.0 s accumulation time was shown to be very sensitive, and the peak width was sharp, so 400.0 s was fixed. Under this condition, the SW amplitude variations were examined. Figure 2B represents the voltammograms ranging from 0.05 V to 0.4 V. The range of amplitude increased very quickly (from 0.05 V to 0.3 V), but slightly decreased to 0.35 V and increased again at 4.0 V. Here, 0.3 V was fixed, and the other parameters were examined under this condition. The results for 400 Hz frequency, −0.8 V accumulation potential, 0.015 V amplitude, and 1.0 V final potential are not shown here. Under these conditions, various analytical-interference ions were examined by adding other metals and analog neurotransmitters into the medium containing 0.1 mg/L EP, with the tenfold spiking of 1 mg/L dopamine, glucose, glycine, acetylcholine, phenylalanine, histidine, and GABA ions. The peak ratios were −12.52%, −1.40%, 3.10%, −3.44%, 14.47%, −1.87%, and 2.71%, respectively. Three analytical interferences were effectively corrected using standard addition methods. The usable working range was examined using SWSV and CV. However, the in vivo condition was not examined.

### 3.3. Statistics, Working Range, and Application

**Figure 3:**
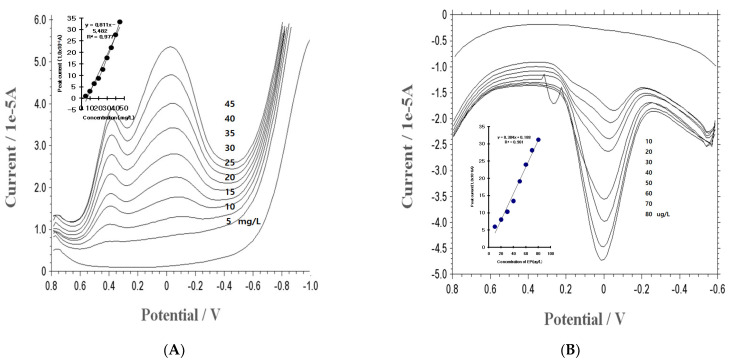
(**A**): SWSV results within the concentration ranges of 5, 10, 15, 20, 25, 30, 35, 40, and 45 mg/L additional EP, using a 30 s accumulation time. (**B**): SWSV results at working ranges of 10, 20, 30, 40, 50, 60, 70, and 80 ug/L additional EP, with a 0.3 V SW amplitude, 400 Hz frequency, −0.8 V initial potential, 0.015 V increment potential, 1 V final potential, and 1 × 10^−4^ sensitivity, were used at a 300 s accumulation time with the BCE.

Using optimized conditions, the detection of the EP concentration within the mg/L and U/L ranges was then evaluated using SWSV. Figure 3A shows the results. The bottom line of the curve is the electrolyte blank solution. The second voltammogram is the result of the additional 5 mg/L, which obtained a small peak at 0.4 V. The third and fourth voltammograms appeared at the 0.4 and 0 V peak currents. Here, the 0.0 V peak current increased linearly from 1.039 to 33.37 × 10^−6^, and the linear curve (not shown) was produced from the regression equation y = 0.811x − 5.482 (y: peak current; x: concentration), R^2^ = 0.977. The anodic and cathodic currents can be applicable in terms of the height range. However, in vivo conditions are needed for pico detection. Here, the accumulation time was increased to 300 s. Figure 3B shows the EP concentration in the ug/L levels by anodic stripping. The SW peak potential of 0 V was obtained, and a linear equation was obtained (y = 0.384x + 0.188), with a relative standard deviation of R^2^ = 0.981. Under these conditions, the detection limit was calculated by 3 *s*/*m*, where *s* is S.D. and *m* is the slope of the calibration plot, and it was found to be 0.45 ug/L (2.45 × 10^−9^ M).

Therefore, the detection limit has been developed to be 10 times or more better than that of existing technologies, as shown in Table 1. These results can be applied to in vivo diagnostic assays and other low detection ranges. For limitless statistics, the chronoamperometric concentration effects were examined.

**Figure 4:**
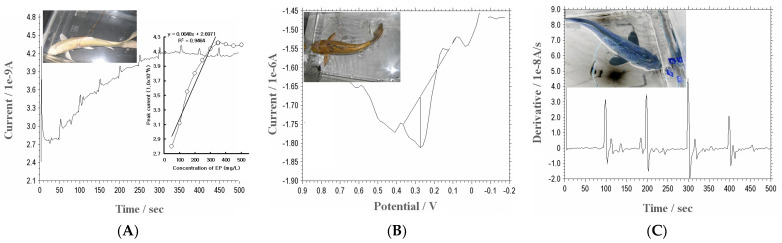
(**A**) EP measurement using a BCE with chronoamperometry at 0.0 V with input of 50 mg/L EP every 50 s. (**B**) Detection of EP concentration in the carp’s caudal fin with SWSV using 400 Hz frequency, −0.8 V accumulation potential, and 1.0 V final potential using the BCE. (**C**) After insertion of the BCE in the right side of the carp’s caudal fin, a stimulus was given every 100 s.

A BCE was used, and EP was measured with a 50 mg/L spike every 50 s. The results are shown in Figure 4A. All optimized conditions were used with 0.2 V initial and high potential, 500 s pulse width, 1 s sample interval, and 1.0 × 10^−7^ A/V sensitivity. The time range was from 0 to 500 s. The electrode signal gradually increased every 50 s and reached 2.8~4.22 × 10^−10^ A. After 350 s, the electrode signal no longer increased and even decreased little by little. Under these conditions, a usable linear equation (y = 0.0048x + 2.697) was obtained as a statistical result for R^2^ = 0.946. For the in vivo assay, a BCE can be made 2.0 mm × 5 cm long so that it can be inserted in in vivo cell systems. After inserting a BCE into the right side of a carp’s caudal fin under anesthesia, the fin’s electrical activity was measured with SWSV using the optimum parameters. Figure 4B shows the practical signal of 1.34 × 10^−7^ A from the right side of the carp’s caudal fin. One peak potential appeared at the 0.2 V potential. 

After it was found that the potential of EP could be measured in a carp’s body muscle, it was applied to other experiments. A BCE was inserted into the right side of a carp’s caudal fin, and the carp was placed in a fishbowl. The left side of the carp’s caudal fin was then given a stimulus every 100 s. The peak currents of EP were measured using the BCE with the Figure 4A conditions. The result is shown in Figure 4C. The time range was from 0 to 500 s. Before 100 s, the electrode signal was close to 0. However, the electrode signal increased dramatically to about 3.1 × 10^−8^ A/s when a stimulus was given at 100 s. After that, the electrode signal dropped rapidly to about −1.6 × 10^−8^ A/s and then rose again. It was close to 0 until the next stimulus was given. The same thing happened every 100 s. A stimulus nerve current was detected in these results, which can be applied in other control systems.

## 4. Conclusions

A three-electrode system was used to detect EP using CV and SWAS. The working electrode was made from a mixture of bismuth and nanotubes at a 1:1 ratio with mineral oil. The analytical conditions were attained at 5–45 mg/L and 10–80 ug/L EP working ranges. The results approximated the nanogram range and reached lower detection limits of 0.45 ug/L (2.45 × 10^−9^ M) compared with other results [6,7,8]. This method was applied to the detection of the right side of a carp’s caudal fin. Unlike other electrodes, a BCE is so tiny that it can be inserted into a carp’s caudal fin without it being destroyed. Therefore, a diagnostic assay can be applied to body fluids and in any other field requiring cell culture and EEG assays, as well as in neuro control.

## Figures and Tables

**Table 1:** Detection limit concentration comparison with existing papers.

This Method: Bismuth Immobilized on a Carbon Nanotube Paste Electrode	0.45 ug/L (2.45 × 10^−9^ M)	References
Graphene-modified glassy carbon electrode	8.9 × 10^−8^ mol L^−1^	[32]
A gold electrode	0.1 μM	[33]
**Glassy carbon electrode modified with catechol**	1.6 µM	[34]
Pt electrode modified by polyaniline–poly(3-methylthiophene)–poly(3,3′-diaminobenzidine)	1.23 × 10^−4^ mmol/L	[35]
Niacin film-coated carbon paste electrode	11.3 nM	[36]
**rGO-AuNPs electrode**	0.45 µM	[37]

## Data Availability

All materials are available from the corresponding author.

## Abbreviations

EP	epinephrine
BCE	bismuth immobilized on a carbon nanotube paste electrode
CE	carbon nanotube paste electrode
SW	square-wave
SWSV	square-wave anodic stripping voltammetry
